# Asthma features in elderly in a terciary outpatients facility

**DOI:** 10.1186/1939-4551-8-S1-A187

**Published:** 2015-04-08

**Authors:** Nathalia Siqueira Robert De Castro, Carolina Tavares De Alcântara, Kelly Yoshimi Kanamori, Jorge Kalil, Bruna Gama Saliba, Rosana Câmara Agondi, Pedro Giavina-Bianchi, Marcelo Vivolo Aun, Carla Bisaccioni

**Affiliations:** 1 Hospital Das Clínicas - University of Sâo Paulo, Brazil; 2 University of São Paulo, Brazil; 3 Brigham and Women's Hospital, Harvard Medical School, USA

## Background

The elderly comprise approximately 10% of the population. Asthma can affect about 10% of these patients; however, the diagnosis is underestimated. The sensitization to at least one aeroallergen in elderly with asthma ranges from 28% to 74%. The aim of this study was to evaluate the characteristics of asthma in patients aged over 60 years, including the frequency of allergic asthma.

## Methods

Cross-sectional study that evaluated 193 elderly patients with asthma followed at an outpatient tertiary service. Patients were considered when asthma met the criteria of GINA 2012 - anamnesis and pulmonary function demonstrating reversibility. These patients were evaluated for gender, current age, age at onset of asthma, lung function and asthma classification. The assessment of atopy was performed by searching for total IgE and specific IgE (*in vivo* or *in vitro*) for house dust mites, molds, cat and dog dander, cockroach and grass.

## Results

Of these 193 patients, 155 (80.3%) were female, the mean age was 69.3 years and the mean age of onset of asthma was 31.8 years. Forty-one patients (21%) had onset of symptoms before 12 years. Spirometry presented mean FEV1 of 64.5%. The classification of asthma showed 75% of patients with severe persistent asthma, 17% with moderate persistent asthma and 8%, mild persistent asthma. In relation to atopy, mean total IgE was 314.3 IU/mL. Positive specific IgE was demonstrated in 70% of patients, although, the group which the age of onset was between 41 and 59 years it was 42%. The majority was sensitized to house dust mites (91% of positive tests).

## Conclusions

In this study, patients whose asthma had an early-onset (≤ 12 years) had more severe outcome. We noted that patients which asthma onset was between 41 to 59 years (25.9% of them) showed a less sensitization to aeroallergens (42% positive). We found also that 31% of elderly patients showed sensitization to molds, compared to literature where the mean was around 20%.

